# Opening Pandora’s Box: Neglected Biochemical Potential of Permafrost-Associated Fungal Communities in a Warming Climate

**DOI:** 10.3390/jof10010020

**Published:** 2023-12-28

**Authors:** Hossein Masigol, Alice Retter, Mohammad Javad Pourmoghaddam, Hossein Amini, Seyedeh Roksana Taheri, Reza Mostowfizadeh-Ghalamfarsa, Mahyar Kimiaei, Hans-Peter Grossart

**Affiliations:** 1 Plankton and Microbial Ecology, Leibniz Institute for Freshwater Ecology and Inland Fisheries (IGB), 16775 Neuglobsow, Germany; alice.retter@igb-berlin.de (A.R.); hossein.amini@igb-berlin.de (H.A.); roxi.thi@gmail.com (S.R.T.); 2 Department of Plant Protection, Faculty of Agricultural Sciences, University of Guilan, Rasht 4199613776, Iran; javad.pormoghadam@gmail.com; 3 Department of Plant Protection, School of Agriculture, Shiraz University, Shiraz 7144113131, Iran; rmostofi@shirazu.ac.ir; 4 Department of Plant Protection, Isfahan (Khorsgan) Branch, Islamic Azad University, Isfahan 3999881551, Iran; mahyar.giah1367@gmail.com; 5 Institute for Biochemistry and Biology, Potsdam University, 14469 Potsdam, Germany

**Keywords:** global warming, permafrost thawing, thermokarst lakes, aquatic fungi, aquatic oomycetes

## Abstract

Permafrost, a vast storage reservoir of frozen organic matter, is rapidly thawing due to climate change, releasing previously preserved carbon into the environment. This phenomenon has significant consequences for microbial communities, including fungi, inhabiting permafrost-associated regions. In this review, we delve into the intricate interplay between permafrost thawing and fungal diversity and functionality with an emphasis on thermokarst lakes. We explore how the release of organic carbon from thawing permafrost alters the composition and activities of fungal communities, emphasizing the potential for shifts in taxonomic diversity and functional gene expression. We discuss the formation of thermokarst lakes, as an example of permafrost thaw-induced ecological disruptions and their impact on fungal communities. Furthermore, we analyze the repercussions of these changes, including effects on nutrient cycling, plant productivity, and greenhouse gas (GHG) emissions. By elucidating the multifaceted relationship between permafrost thaw and aquatic fungi, this review provides valuable insights into the ecological consequences of ongoing climate change in permafrost-affected regions.

## 1. Introduction

**Figure 1:**
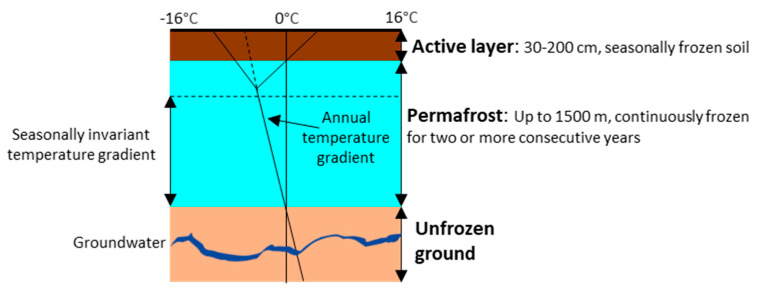
The permafrost vertical landscape showing an active layer above and unfrozen ground below. Temperatures typically range from 5 °C in the active layer to −12 °C within the permafrost. The black line at the top corresponds to temperature variation in the permafrost.

Climate change is defined as long-term shifts in temperatures and weather patterns as a result of human activities, especially the burning of fossil fuels since the industrial revolution in the 1800s [1]. Permafrost is soil/underwater sediment which remains below 0 °C for two years or more, overlain by an active layer above and unfrozen ground below (Figure 1) [2]. Permafrost thaw can lead to, e.g., land subsidence, habitat disruption, changes in vegetation, and altered hydrological interactions, implying major ecological impacts on the biogeochemistry of permafrost [3,4].

Nevertheless, understanding how permafrost thaw might alter biodiversity and subsequent functioning of microbial communities in permafrost is largely ignored. This is a matter of great importance as any shift in diversity and functionality of prokaryotic and eukaryotic (e.g., fungal) communities might cause more serious large-scale ecological feedback loops, e.g., via changes in atmospheric GHG emissions.

Permafrost contains a substantial amount of organic carbon (ca. 1400 to 1850 gigatons) in the form of dead plants, animals, and microbes that have been preserved in the frozen ground for thousands of years [5]. This organic carbon is essentially “locked” in the permafrost, as cold temperatures and lack of oxygen greatly inhibit microbial decomposition. As permafrost keeps thawing, however, this organic carbon reservoir will be gradually available for the microbial community [6]. Competition over these newly thawed carbon sources will begin to alter the diversity of microbial groups as some taxa might benefit from the newly released organic matter more than others [7,8]. One could envision scenarios in which some taxa with specific functional capabilities will dominate the community in response to altered carbon dynamics caused by permafrost thaw. In addition, physical disturbances have the potential to change community network dynamics and interactions of distinct microbial taxa in different permafrost-associated horizons [9,10,11]. For instance, more frequent thawing events could lead to changes in hydrological interactions between frozen soil, and unfrozen (e.g., groundwater-bearing subsurface) water within and beneath the permafrost layers. This potentially leads to changing flow paths and distribution of water within and between the permafrost and adjacent layers, regulating energy (nutrient, and carbon) fluxes, and ongoing permafrost degradation in these systems. Different layers experience higher degrees of permafrost devolution, rendering them less distinct. This might significantly impact the already established existence of intricate interactions among specific microbial communities within each horizon.

The purpose of this paper is to give an overview of the recent research on the alterations in and effects on organic matter (OM) degradation in permafrost by focusing on the largely neglected fungal communities. We use aquatic fungi in thermokarst lakes as an example to evaluate effects of permafrost thawing on fungal biodiversity and their involvement in OM turnover and other biotic or abiotic processes, and thus carbon and greenhouse gas dynamics.

## 2. A Case in Point: Thermokarst Lakes, a Geo-Ecological Change Caused by Permafrost Thaw

**Figure 2:**
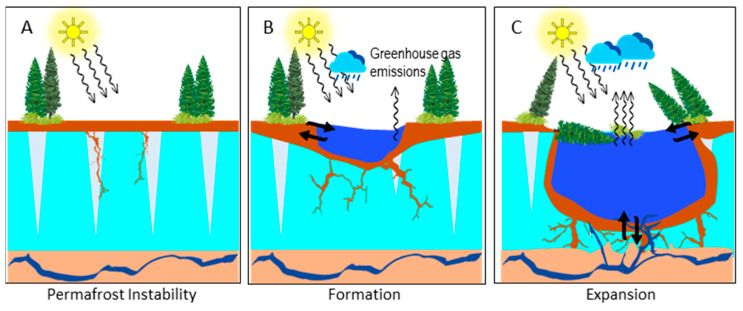
Schematic figure of the different stages in thermokarst lake formation and ecosystem succession. (**A**) Due to climatic change (e.g., climate warming) permafrost degradation is initiated. (**B**) The collapsing ground can give rise to collection sites for precipitation and snow melt (i.e., thermokarst lakes) which further accelerates permafrost devolution and initiates biogeochemical processing of organic matter (OM) (e.g., carbon transformation) and thus enhances greenhouse gas emissions. (**C**) Advanced stage of thermokarst expansion facilitating the deterioration of the adjacent permafrost ecosystem. This can lead to an even larger turnover of OM (e.g., primarily stored in the active layer and continuously thawing permafrost) and possible changes in the hydrological exchange with heretofore confined groundwater.

As stated above, thawing has the potential to destabilize permafrost, its complex network of microbial inhabitants, and its interaction with the active layer above and unfrozen ground below [12]. The formation of thermokarst lakes is an important example of such permafrost thaw-derived ecosystem changes. Thermokarst lakes are formed when permafrost thaws due to rising temperatures, causing the ground to collapse and creating depressions that fill with water mainly originating from precipitation and snowmelt, but potentially also from groundwater (e.g., endo-permafrost groundwater forming under warming conditions) [13] (Figure 2). These lakes are critically important in the context of global climate change because they present major sources of atmospheric GHG emissions [14]. Their expansion, both vertically and horizontally, further intensifies this impact, and they alter local hydrology to enhance methane production in lake sediments [15].

Ecologically speaking, thermokarst lakes have the potential to turn permafrost into a hotspot for unexpected taxonomic and functional diversification of microbial communities. In fact, they are reservoirs for microbial communities from permafrost soils which have survived in subzero temperatures for perhaps thousands of years. These communities had to adapt to extreme conditions such as high salinity, low water and nutrient availability, anoxia, and low pH [16]. As permafrost thaws, they are transported into surrounding ecosystems, such as thermokarst lakes. Here, they encounter more favorable and stable environmental conditions, leading to major ecological consequences. For example, thermokarst lakes which currently cover over one million km^2^ receive considerable input of both labile and refractory dissolved organic matter (DOM) from thawing permafrost. To grasp the scale of such a phenomenon, prokaryotic and eukaryotic communities in these lakes could potentially recycle permafrost-originated OM (including DOM), generate CO_2_ and CH_4_, and contribute to releasing up to ca. 14–18 Tg C per year [17], turning these lakes into massive hotspots of GHG emissions [18]. Therefore, thermokarst lakes could initiate a major positive climate feedback by releasing large quantities of long-term carbon stocks as GHG with high warming potentials into the atmosphere.

## 3. The Vital Role of Fungi in Permafrost Ecosystems

Fungi actively contribute to carbon cycling in a wide range of ecosystems, including permafrost soil [19]. In fact, the presence and viability of fungal taxa in permafrost soil have been shown using both culture-dependent and culture-independent methods [20]. They are expected to be in a state of survival exhibiting low levels of activity as they are exposed to extreme environmental conditions such as low oxygen levels and subzero temperatures. However, as permafrost thaws, fungi can potentially become active decomposers of previously frozen OM, contributing to GHG emissions (Figure 2), and thereby amplifying positive climate change feedback loops. At the same time, the diversity and ecological functioning of fungi could be strongly impacted by the fast-paced alterations and disturbances in permafrost ecosystems. For instance, permafrost might contain various niche habitats that harbor specific groups of fungi, well-adapted to the predominant conditions, and thereby secure their intricate interactions with other prokaryotic and eukaryotic communities. As permafrost keeps thawing, the established local fungal assemblages in permafrost will be disrupted. It is noteworthy to clarify that the amplitude of such disruptions is not limited to permafrost itself. As a result of thawing, the active layer and frozen ground will be physiochemically altered as well, resulting in fungal community shifts in terms of their distribution and function.

The formation of thermokarst lakes is a major disruption in which the zonation of permafrost will be fully/partly degraded and fungal communities could thus be dislocated and end up exposed to changed environmental conditions.

Consequently, these alterations will be reflected in the diversity and ecology of the fungal community in thawing permafrost soils. In fact, constant environmental changes will force fungal taxa to compete under new evolutionary pressures which might change their functions and community composition. For example, as temperature increases, more refractory OM, e.g., lignin-derived, will be available in permafrost, creating a suitable niche for those fungi which can degrade these high molecular weight compounds effectively. More importantly, ecosystem-scale shifts will be imminent as most fungal taxa are simultaneously interacting with various trophic levels. For example, plants in permafrost benefit from mycorrhizal fungi for nutrient uptake, stress tolerance, and disease resistance. In the absence of their fungal symbionts, these plants growing on the active permafrost layer might not be able to compete and become a subdominant species.

## 4. How Permafrost Thawing Will Affect Fungal Diversity?

Our knowledge regarding the diversity of fungi in permafrost-associated habitats relies mainly on culture-independent techniques. In almost all studies, *Ascomycota* has been the most dominant phylum followed by *Basidiomycota* and *Chytridiomycota* (Table 1). Additionally, it has been shown that fungal community composition strongly responses to abiotic and biotic alterations in permafrost. However, with a few exceptions, a higher resolution to the genus level is usually missing which makes further ecological interpretations hard to achieve.

### 4.1. Relationship between Fungal Diversity and DOM Turnover

Most recently, Hu et al. [22] studied 196 thermokarst lakes at 48 sites along a 1100-km permafrost transect across the Tibetan Plateau to understand the effect of sunlight on microbial degradation of DOM. They found that sunlight promoted microbial (in particular fungal) degradation by converting high molecular weight aromatic-like DOC into more biodegradable DOC. In their study, Eurotiomycetes, Tremellomycetes, and Dothideomycetes, were the most dominant groups, accounting for 12, 10, and 9% of the whole fungal community, respectively. Importantly, the authors showed that more than 65% of these fungal taxa were associated with a saprophytic lifestyle. This is consistent with the finding that saprotrophic fungi are equipped with different nonspecific oxidizing enzymes such as laccases, lignin and manganese peroxidases which make them able to degrade/transform a variety of DOM with different degrees of bioavailability [31].

Additionally, Kluge et al. [32] investigated fungal diversity in thermokarst lakes from Alaska, Greenland, Canada, Sweden, and Western Siberia. These sites represented the highest to lowest levels of permafrost degradation, respectively. Kluge et al. [32] addressed whether there was a relationship between DOM quality and availability and fungal community structures and identified several compositional and functional trends. First, they showed that fungal communities became more homogeneous with the degree of DOM degradation, resulting in a significant loss of taxonomic diversity. Similarly, Chen et al. [25] conducted two incubation experiments to explore changes in fungal communities of permafrost soils when exposed to 5 °C for 11 days. Likewise, Kluge et al. [32] experimentally showed that permafrost thaw caused a significant loss in fungal taxonomic diversity. These shifts will probably continuously happen as these communities are increasingly transported from permafrost soils into thermokarst lakes. Secondly, it was shown by Kluge et al. [32] that the DOM quality covaried with fungal community composition. In other words, while some OTUs thrive in the presence of labile DOM and nutrients (in the early stages of thermokarst lake formation), others benefit most from more recalcitrant compounds (representing the late stages of thermokarst lake formation). Additionally, Wurzbacher et al. [28] studied fungal communities of emerging, middle-aged, and old permafrost thaw ponds in Canada. In their study, emerging ponds had higher refractory DOM than older ones. At the same time, they could observe a successional progression of thaw ponds in terms of their relative abundance of fungal taxonomic groups. They showed that fungal community composition as well as composition of carbon compounds were subject to a progressive temporal succession. For example, the abundance of fungal taxa associated with Basidiomycota was significantly higher in older ponds, whereas Rozellomycota were dominating in emerging ponds.

### 4.2. Fungal Communities and Other Abiotic Parameters

In addition to DOM, other physico-chemical parameters of permafrost have shown to play a role for the diversity of fungal communities. One example is the study conducted by Jiang et al. [21] who investigated the composition and diversity of fungal communities in permafrost from the Greater Xing’an Mountains. They conducted a warming experiment to evaluate how fungal communities would be altered if soil samples from permafrost were exposed to 0 °C, 2 °C, and 4 °C compared to −2 °C, which is the natural temperature of permafrost soil. Firstly, they observed that most soil parameters were significantly changing during their warming experiment. Secondly, they revealed that there was a significant correlation between fungal communities of each treatment group with some distinct physicochemical parameters. For instance, at 4 °C of warming, fungal abundance was positively correlated with carbon and available phosphorus but negatively with soil organic carbon, available nitrogen, and pH content. Shifts in fungal diversity are therefore a relatively quick response to the fast-paced physico-chemical transformation of permafrost thaw.

The composition of fungal communities was studied in relation to four soil horizon types across different types of permafrost-affected soil in Western Canadian Arctic [24]. These horizon types include upper topsoil, cryoturbated soil OM (cryoOM), mineral subsoil, and permafrost. These horizons and permafrost were different in terms of dissolved organic carbon (DOC), total carbon (C_tot_), total nitrogen (N_tot_), and C/N ratio. They showed that different soil horizons had a significant effect on fungal community composition. For instance, while the genera *Lachnum* and *Phialocephala* were relatively more abundant in topsoil, *Russula* accounted for a larger proportion in cryoOM compared to other horizons. Based on trophic modes, ectomycorrhizal and wood saprotrophs had a significantly higher mean proportion in cryoOM compared to topsoil, while the proportion of pathotrophs was significantly lower in topsoil. They also showed distinct correlation patterns between generalist and specialist fungal taxa with environmental factors from different soil horizons, with their topological roles shifting between different horizons. This study highlights the delicate relationship between fungal communities and other abiotic factors in permafrost-affected soils.

Sannino et al. [23] investigated the impact of abiotic factors on fungal diversity from permafrost in the Italian Central Alps along a depth gradient (410 to 524 cm from the surface). The most prevalent fungal phyla along the depth gradient were *Ascomycota* and *Basidiomycota*, respectively. In particular, the genus *Meyerozyma* (*Ascomycota*) always exhibited an abundance > 40%. Interestingly, positive co-occurrence patterns were observed among fungal genera (e.g., *Meyerozyma* vs. *Exidia*) and between bacterial and fungal genera (e.g., *Acinetobacter* vs. *Alternaria* and *Vishniacozyma*). The depth gradient was the only abiotic factor significantly impacting fungal diversity. It was shown that while diversity was higher in deeper layers, evenness decreased. Results from this study highlight once again the ecological and taxonomic balance of fungal communities within permafrost ecosystems which could be dramatically impacted by reducing permafrost extent and thickening of its active layer caused by global climate warming.

Also, Bomberg et al. [26] studied several lakes in the Kangerlussuaq area (situated on the west coast of Greenland) and showed members of *Chytridiomycota* to be the most common taxa. They also showed that fungal communities occurring in lakes were not affected by organic or inorganic carbon quantities but by salinity and sulfate concentrations. This study is another great example of the transition of fungal communities from thawing permafrost soils to specific niches in thermokarst lakes. As an example, the saprobic fungus *Ramicandelaber* (Zygomycetes *sensu latu*) constituted a major portion of the fungal community in one of thermokarst lakes. The studied lake must have received constant loads of organic material from the surrounding terrestrial habitats which eventually led to the introduction of the soil-related fungus *Ramicandelaber* into the lake. During the aging of the pond, a progressive succession takes place in which the taxon *Ramicandelaber* might get outnumbered by newcomers which are better adapted to the changing conditions of the lake.

## 5. How Permafrost Thawing Will Impact Ecological Functions/Interactions of Fungi?

### 5.1. Relationship between Fungal functions and DOM Turnover

Thermokarst lakes can be considered as high DOM turnover ecosystems in the sense that species composition, community structure, and ecological interactions are subject to rapid and dynamic changes. Changes in the quality and quantity of DOM are important for microbial evolution in thermokarst lakes. Accordingly, the functional potential and related ecological roles of newly formed communities will be impacted too. Therefore, it can be hypothesized that the abundance of fungal functional genes associated with transformation/degradation of DOM will be altered as thermokarst lakes age.

Chen et al. [25] not only showed a loss in taxonomic diversity of fungal communities as permafrost thaws, but also observed a shift in the abundance and higher expression of functional genes involved in the degradation/transformation of DOM as the lake matured. In the beginning of the thawing process, functional genes associated with the degradation of starch as a labile source of carbon had the highest abundance. However, functional genes which could transform/degrade aromatics as refractory carbon sources dominate when thermokarst lakes have matured. Zhong et al. [33] also collected sub-permafrost groundwater samples from the Qinghai-Tibet Plateau (QTP) and showed that permafrost degradation increases potential functions of fungi relevant to carbon metabolism. Similarly, Kluge et al. [32] investigated the relationships between CAZymes (a group of enzymes responsible for fungal degradation and transformation of organic matter) and DOM quality in the water. They observed a positive correlation between the composition of CAZymes and DOM, showing the impact of DOM quality on fungal functional diversity in thermokarst lakes.

Another piece of evidence regarding shifts in the composition of fungal functional genes stems from Cheng et al. [27], who analyzed the effects of winter warming on fungal communities in thawing-induced permafrost soil in the Alaskan tundra. They showed that the overall functional gene composition of fungal communities was significantly altered by warming, even though their composition remained unchanged. They further suggested that higher relative abundances of functional genes associated with carbon degradation (such as invertase, xylose reductase, and vanillin dehydrogenase) are closely associated with the positive feedback of fungal communities to a warmer climate.

The same trend was observed by Coolen and Orsi [34] who compared the transcriptional response of the permafrost microbial community before and after 11 days of permafrost soil thaw. While stress responses, survival strategies, and maintenance processes were dominant under frozen conditions, a sharp enzymatic response in the transformation/degradation of permafrost soil organic matter was observed upon thaw. Some bacterial groups as well as ascomycete fungi were among the groups with the largest transcriptional response upon thaw. For example, hydrolyses by ascomycete fungi (e.g., glycosylases, peptidases, etc.), involved in the initial degradation of complex permafrost soil biopolymers, were increasingly expressed after 11 days of thaw.

Additionally, Coolen et al. [35] conducted time-series incubation experiments with Holocene permafrost soils in Alaska at 4 °C for up to 11 days. During this period, changes in the activity of exoenzymes were determined as these enzymes provide information on the actual bioavailability of the naturally available substrates in different layers of permafrost soils. The study revealed that recalcitrant soil organic matter (SOM) was more abundant in deeper and older permafrost horizons (>49 cm). Interestingly, more than 83% of all OTUs associated with basidiomycetes were exclusively recovered from these freshly thawed layers after 6 or 11 days of thaw. Therefore, Coolen et al. [35] suggested that basidiomycetous fungi associated with deeper permafrost soil layers could be one of the main groups capable of degrading recalcitrant SOM, thereby releasing substrates for bacterial communities. As permafrost gradually collapses, a mixture of both fungal communities and recalcitrant and/or labile SOM are transported into thermokarst lakes. Depending on the fate of SOM in the thermokarst lakes, the rate of fungal exchanges and functions can substantially vary under different scenarios, e.g., fluctuations in environmental parameters, in particular temperature. This is a major driver of functional diversification in fungal communities following permafrost thaw.

Wu et al. [36] deployed a 5-year warming treatment and investigated shifts in the functional gene array of fungal communities at an Alaska tundra site and their results partially contradict the above-mentioned studies. Although the authors showed that the metabolic capacity of fungal communities associated with C metabolism increased under warming, this was not the case in the deeper organic layers of the permafrost soil. For example, the signal intensity of genes encoding for phenol oxidase involved in aromatic component decomposition had decreased in the deep layer by 10%. This suggested a reduced C-fungal degrading capacity in this deep layer, presumably related to fungal communities trying to alleviate their energy spent via increased respiration. These results underline the difficulty in understanding the ecological role of fungi during thermokarst lake succession. In other words, movement of fungal communities with altered functional capacities from the different permafrost soil layers into thermokarst lakes renders it challenging to predict the actual biochemical potential of these lakes in different warming scenarios. Another similar study was performed by Romero-Olivares et al. [37] in permafrost regions of Alaska by investigating soil fungal meta-transcriptomes under long-term experimental warming and drying. Firstly, they showed a significant increase in fungal orders such as *Arthoniales* and *Xylariales* which also include taxa with pronounced stress-tolerant traits. Secondly, observed ecological tradeoffs in fungal resource allocation were a result of warming favoring cell metabolic maintenance over decomposition. In fact, under warming, metabolic clusters of orthologous genes (COGs) (associated with maintenance) were transcribed more significantly than carbohydrate-active enzymes (CAZy) (associated with carbohydrate decomposition).

### 5.2. Ecosystem-Scale Shifts by Fungal Communities

Changes in fungal communities and their functions have the potential to mediate far-reaching ecological responses to permafrost thaw. In fact, these responses might lead to ecosystem-scale shifts in the fauna and flora of permafrost-associated ecosystems. For example, nutrient cycling dynamics and plant productivity are two major proxies for which large-scale impacts of fungal communities on permafrost-associated regions could be observed.

Nutrient cycling in the permafrost is already significantly impacted by climate change. For example, as permafrost keeps thawing, more nitrogen will be released, stimulating plant productivity. Since mycorrhizal fungi are thought to facilitate plant access to nutrients, Hewitt et al. [38] investigated whether they promote nutrient accessibility to plants in freshly thawed permafrost. They aimed to examine whether fungi could contribute to vertical transfer of nitrogen from thawed permafrost to host plants. Firstly, they observed that the overall connectivity of fungal OTUs from the thaw front to the roots front of four different plant species is ubiquitous. Secondly, the presence of five and three ascomycete and basidiomycete OTUs were shown to be linked to increased plant access to newly thawed permafrost-derived nitrogen. Finally, they concluded that the contribution of fungi into the vertical redistribution of deep, permafrost-derived nutrients can be considered as an essential mechanism for the whole permafrost ecosystems. By such a contribution as this, fungal communities might alleviate nitrogen limitation and stimulate productivity in future warmer tundra regions. These findings can explain the results from Hewitt et al. [39], who showed that, in contrast to non-mycorrhizal plants, mycorrhizal ones don’t extend their roots to the thaw front. Therefore, it can be argued that mycorrhizal plants are associated with mycobionts that occur at the permafrost thaw front and make resources available for plant roots in the rhizosphere zone.

Another great example is provided by Schütte et al. [40] who evaluated the potential of fungal community composition of an intact permafrost plateau forest soil and an adjacent thermokarst bog in Alaska to mediate shifts in plant composition. They observed a significant shift in the fungal community composition between thawed and intact permafrost sites, with a higher abundance of putative fungal pathogens and mycorrhizal fungal taxa in thawed vs. intact permafrost sites, respectively. They also grew local plants in a greenhouse under sterile conditions and soils containing soil microbial inoculum from the rooting zone of the permafrost plateau and the thermokarst bogs. Interestingly, productivity of plant species in the soil inoculated using microbial inoculum from the thermokarst bogs were the lowest, suggesting an overall negative effect of the microbial community present at the permafrost thawed sites. Therefore, fungal communities in thermokarst bogs have a crucial potential to control plant community composition and their development in thawing permafrost soils.

## 6. Conclusions

In this review paper, we discussed the impact of permafrost thaw on fungal communities and their potential ecological roles by addressing the prominent example of temporal succession of thermokarst lakes. We highlighted the significance of permafrost as a reservoir of organic carbon and carbon release under thawing conditions, presumably altering taxonomic and functional capacities of extant fungal communities. For this, we focussed on the formation of thermokarst lakes in permafrost and resulting implications for fungi as important players under ongoing climate change. We found that thawing permafrost is often associated with dynamic physicochemical changes, entailing functional and compositional shifts. Moreover, in many cases fungal diversity was lost during thermokarst lake succession, though occasionally rare taxa can emerge.

There are still major gaps which limit our understanding on the amplitude of such changes. To date, most permafrost regions have remained under- or unstudied, which greatly hampers our attempt to characterize local specificity in fungal communities. Additionally, it remains unclear how fungi contribute qualitatively and quantitatively to the turnover of carbon during permafrost thawing. In this context, implementing DNA-based approaches, such as metagenomics and metatranscriptomics will allow us to accurately screen fungal diversity and activity in less-thawed permafrost regions and compare those to areas heavily influenced by permafrost soil thawing. Limitations in current knowledge are to a large extent due to the limited use of accurate and quantitative methods such as isotope tracing which allow to evaluate how fungi can agitate energy flow in thawing permafrost soils. Such methods will allow us to develop and address relevant hypotheses on expected consequences for fungal community and activity dynamics as well as their feedback to global carbon and greenhouse gas cycling in a warming world.

## Figures and Tables

**Table 1:** Major studies conducted on the diversity of the fungal community in permafrost-associated regions.

Study	Location and Habitat	Dominant Phyla/Taxa (Respectively) *	Important Features
Jiang et al. [21]	Greater Xing’an Mountains, China, permafrost	*Asco*, *Basidio*, *Mortier*, *Rozello*	Shifting fungal communities in a permafrost thawing experiment
Hu et al. [22]	Tibetan Plateau, thermokarst lakes	*Asco*, *Basidio*	With mainly saprophytic taxa
Sannino et al. [23]	Scorluzzo active rock glacier, Italian Central Alps, permafrost core	*Asco*, *Basidio*	Cooccurrence of fungal taxa with certain bacteria
Varsadiya et al. [24]	Herschel Island, Beaufort Sea, Canada, Soil horizons of the active layer and permafrost	*Asco*, *Basidio*, *Mortier*	Topological role shifts in taxa
Chen et al. [25]	Tibetan Plateau, permafrost	*Ascoa*, *Basidi*, *Chytridi*, *Glomero*, *Rozello*, *Zygo*	Decreasing alpha-diversity after a permafrost thawing experiment
Bomberg et al. [26]	Kangerlussuaq area west coast of Greenland, pond/lake samples, ice and melt water samples	*Basidio*, *Asco*, *Chytridio*	Fungal communities were affected by salinity and sulfate concentrations
Cheng et al. [27]	Alaska, soil samples from a permafrost-associated tundra	*Asco*, *Basidio*, *Chytridio*, *Blastocladio*	Winter warming increases carbon degradation capacities of fungal communities
Wurzbacher et al. [28]	Canada, thaw ponds from sporadic permafrost areas	*Asco*, *Basidio*, *Chytridio*	Dominance of unknown fungal taxa in emerging, middle-aged, and old ponds
Frey et al. [29]	Mountain ridge ‘Muot da Barba Peider’, eastern Switzerland, soil from active and permafrost layers	*Asco*, *Basidio*, *Zygo*, *Chytridio*	Dominance of lichenized taxa and cold-adapted yeasts
Penton et al. [30]	Alaska, soils from permafrost and prairie	*Asco*, *Basidio*, *Chytridio*	Significant effects of sample depth on fungal communities

* Abbeviations: *Asco*, *Basidio*, *Chytridi*, *Glomero*, *Mortier*, *Rozello*, *Zygo*: Ascomycota, Basidiomycota, Chytridiomycota, Glomeromycota, Mortierellomycota, Rozellomycota, and Zygomycota, respectively.

## Data Availability

All relevant data generated or analyzed during this study are included in this manuscript.
